# Stroke risk associated with balloon based catheter ablation for atrial fibrillation: Rationale and design of the MACPAF Study

**DOI:** 10.1186/1471-2377-10-63

**Published:** 2010-07-21

**Authors:** Karl Georg Haeusler, Lydia Koch, Juliane Ueberreiter, Matthias Endres, Heinz-Peter Schultheiss, Peter U Heuschmann, Alexander Schirdewan, Jochen B Fiebach

**Affiliations:** 1 Department of Neurology, Charité - University Medicine Berlin, Germany; 2 Center for Stroke Research, Charité, Berlin, Germany; 3 Department of Cardiology and Pneumology, Charité - University Medicine Berlin, Germany

## Abstract

**Background:**

Catheter ablation of the pulmonary veins has become accepted as a standard therapeutic approach for symptomatic paroxysmal atrial fibrillation (AF). However, there is some evidence for an ablation associated (silent) stroke risk, lowering the hope to limit the stroke risk by restoration of rhythm over rate control in AF. The purpose of the prospective randomized single-center study "Mesh Ablator versus Cryoballoon Pulmonary Vein Ablation of Symptomatic Paroxysmal Atrial Fibrillation" (MACPAF) is to compare the efficacy and safety of two balloon based pulmonary vein ablation systems in patients with symptomatic paroxysmal AF.

**Methods/Design:**

Patients are randomized 1:1 for the Arctic Front^® ^or the HD Mesh Ablator^® ^catheter for left atrial catheter ablation (LACA). The predefined endpoints will be assessed by brain magnetic resonance imaging (MRI), neuro(psycho)logical tests and a subcutaneously implanted reveal recorder for AF detection. According to statistics 108 patients will be enrolled.

**Discussion:**

Findings from the MACPAF trial will help to balance the benefits and risks of LACA for symptomatic paroxysmal AF. Using serial brain MRIs might help to identify patients at risk for LACA-associated cerebral thromboembolism. Potential limitations of the study are the single-center design, the existence of a variety of LACA-catheters, the missing placebo-group and the impossibility to assess the primary endpoint in a blinded fashion.

**Trial registration:**

clinicaltrials.gov NCT01061931

## Background

About 20-37% of all ischemic strokes are of cardioembolic origin [1,2], caused by different cardiac disorders, but most commonly by atrial fibrillation (AF). The current demographic trends will lead to an increasing prevalence of AF [3,4] and implicate a growing risk of cardioembolic stroke [1]. In general, cardioembolic stroke has a worse prognosis and a higher recurrence rate than other ischemic stroke subtypes [5]. According to recent guidelines, the left atrial catheter ablation (LACA) of pulmonary veins (PV) is approved for treatment of symptomatic atrial fibrillation (AF) in patients with paroxysmal or persistent AF, refractory to antiarrhythmic medication [6]. Within the last years, LACA using radiofrequency has become a routine procedure and is widely used [7]. In patients with paroxysmal AF (<65 years) and without structural heart disease the achieved rate of persisting sinus rhythm is best and varies between 60% and 80% [8,9]. Major complications like pericardial tamponade, PV-stenosis, atrial-esophageal fistula, injury of the phrenic nerve and vascular damage by atrial and venous sheaths were detected more often in older patients (≥75 years) and patients with congestive heart failure [10]. Furthermore, there is some evidence for an ablation associated stroke risk [9,11,12], lowering the (so far) unproven hope to limit the AF-associated stroke risk by LACA. Considering that most AF patients "eligible" for LACA have a low stroke risk [13], the evident stroke rate of 0.4-1.1% in several registries [9-12] and a "silent" stroke rate of 10-11.3% [14,15] raise concerns and afford further investigation.

As demonstrated in humans, a balloon catheter using cryoenergy (Arctic Front^®^, Medtronic, Inc.) had safety advantages compared to radiofrequency devices [16]. Another basket shaped catheter device (HD Mesh ablator^®^, C.R. Bard, Inc.) combines circumferential mapping and radiofrequency delivery, leading to a reduced procedure and fluoroscopy time [17]. Both catheters are already used in daily clinical practice at the Department of Cardiology, Charité Campus Benjamin Franklin (CBF). Therefore, we designed a randomized single center study to evaluate the risks and benefits of LACA, using these innovative catheter devices.

Conducting MACPAF, we focus on the devices' periprocedural (silent) stroke rates and subsequent neuro(psycho)logical deficits. By using a highly standardized monitoring including serial brain magnetic resonance imaging (MRI) and neuro(psycho)logical tests we hope to set new standards for further trials in this regard. Furthermore, the implanted reveal recorder Reveal XT^® ^(Medtronic, Inc.) is highly accurate to detect clinically silent AF episodes, not infrequently occurring after LACA [18] on the one hand, and to determine the AF burden, on the other hand.

## Methods/Design

### Study population and study design

The Mesh Ablator versus Cryoballoon Pulmonary Vein Ablation of Symptomatic Paroxysmal Atrial Fibrillation (MACPAF) study is designed as a prospective randomized single center study conducted by the Department of Cardiology and Pneumology (Charité, CBF), the Center for Stroke Research Berlin and the Department of Neurology (Charité, CBF). The study has been approved by the local Ethics Committee (EA4/087/08) in February 2009. Randomization started in April 2009. Study entry and storage of data require subsequent consent by the patient. The inclusion and exclusion criteria are summarized in Table 1. Before undergoing LACA, patients receive an implantable device (Reveal XT^®^, Medtronic, Inc.) able to record the AF burden for at least 2 years. Within 24 h before LACA, all patients undergo transesophageal echocardiography (for exclusion of atrial thrombi), a brain MRI as well as a neuro(psycho)logical examination. A repeat brain MRI and neuro(psycho)logical examination will be performed within 48 h after the procedure. Patients will be randomized 1:1 for the cryoenergy device (Arctic Front^®^) or the newest device using conventional radiofrequency (HD Mesh ablator^®^). Before PV ablation, a CT image will be performed and segmented by CARTO Merge^® ^(Biosense Webster, Inc.) for visualization of PV anatomy. The LACA procedure will be performed by first introducing a ring shaped decapolar catheter via a single transseptal puncture. Afterwards, this catheter will be exchanged for the cryoballoon catheter or the mesh ablator, respectively. Finally, PV conduction will be re-evaluated by the decapolar catheter. In case of remaining PV conduction, the ablation procedure will be continued until isolation of all PVs is achieved. After transseptal puncture, heparin will be given to assure an activated clotting time of >300 s. Follow-ups will take place at 1, 3, 6, 9 and 12 months after LACA, including data acquisition from the Reveal XT^® ^or a 5-day-holter-ECG, respectively. Brain MRI and neuro(psycho)logical examination will be repeated after 6 months. The patient will be put on oral anticoagulation (INR 2-3) for at least 6 months after LACA to improve comparability of the results. The end of study is expected for late 2011.

**Table 1:** MACPAF: Inclusion and exclusion criteria.

**Inclusion criteria**:
• Patients with ECG-documented symptomatic paroxysmal atrial fibrillation • At least one ineffective antiarrhythmic drug treatment • Age ≥ 18 and ≤ 75 years

**Exclusion criteria**

• Previous ablation procedure for AF • Cardiac surgery ≤ 3 months • Left atrial diameter > 50 mm or ejection fraction < 35% • Instable coronary artery disease or clinically relevant cardiac valve insufficiency • Hyperthyroidism, pregnancy or lactation • Coumadin or heparin intolerance • Concomitant disease with expected lifespan < 2 years • Contraindications for MRI • Acute cerebral infarction (DWI lesion) detected by brain MRI before LACA

### Aims and objectives

The primary objective of MACPAF is the efficacy of PV isolation, defined by achieving an exit block for all PVs per patient. We hypothesize that the cryoballoon catheter is more effective compared to the mesh ablator catheter in this regard. This is the only objective, which can not be assessed in a blinded fashion for the used ablation catheter. The secondary objective is the detection of LACA-associated (silent) cerebral thromboembolism by brain MRI and by neuro(psycho)logical examination. Further objectives are the determination of the AF recurrence rate after LACA using an implantable device, the detection of non-neurological major complications after LACA and the detection of (silent) cerebral thromboembolism within 6 months after LACA.

### Sample size calculation and Statistical analysis

The primary endpoint for a patient is achieved when all four pulmonary veins are isolated. We hypothesize that corresponding to this primary endpoint the Arctic Front^®^ catheter is superior to the HD Mesh Ablator^®^ catheter. The null hypothesis of equal proportions of successful outcomes for both methods will be tested by the two-sided Fisher's exact test at significance level 0.05. Assuming a proportion of 75% successful ablations for the HD Mesh Ablation^®^ catheter, sample sizes of 54 for both treatment groups achieve 80% power to detect a 20% greater proportion of successes for the Arctic Front^®^ catheter. This corresponds to a proportion of 95% successful ablations for the Arctic Front^®^ catheter - a relative improvement of 27% for the Arctic Front^®^ catheter in comparison to the HD Mesh Ablator^®^ catheter.

An intention-to-treat analysis will be used to analyze treatment differences concerning predefined outcome measures. Treatment allocation will be done by gender-stratified randomization. For all outcome variables descriptive statistics will be collected. For quantitative traits, we will compute absolute and relative frequencies. In the case of continuous or quasi-continuous variables with nearly symmetric distribution, we will compute arithmetic mean, standard deviation, minimal and maximal values, otherwise median, quartiles, minimal and maximal values. Fisher's exact test will be used to compare proportions for dichotomous outcomes between independent groups or to test independency of two dichotomous traits within a population. Hypotheses about proportions for dichotomous outcomes within a single group (one sample analysis) will be tested by the binomial test. Assuming equal shaped distributions in the two treatment groups for at least ordinal scaled outcome variables group specific location parameters will be compared by the t-test or the Mann-Whitney test, respectively, depending on normality or non-normality of the distribution. Analysis regarding time to first AF-recurrence: Defining 'no observed AF-recurrence between 3 months after LACA and the end of follow-up' as 'survival', survival functions will be estimated by the Kaplan-Meier method. The null hypothesis of equal survival curves for the two treatment groups will be tested by the Log-Rank test.

### MRI analysis

MRI examination is performed if the patient is able to give informed consent. Using a 3T MR scanner (Tim Trio; Siemens AG, Erlangen, Germany) the following sequences will be done 24 hours before as well as 24-48 hours after the ablation procedure: T2*-weighted imaging to screen for intracranial hemorrhage; diffusion-weighted magnetic resonance imaging (DWI) to assess cerebral infarction; Fluid-attenuated inverse recovery (FLAIR) to estimate microangiopathic lesions load and to investigate the age of recent lesions; time-of-flight MR-Angiography (TOF MRA) to detect vessel stenosis or occlusion. Six month after LACA a third measurement without TOF MRA will be performed to assess the incidence of (silent) strokes by time. For sequence parameters see [19]. Blinded MRI reading will be done by a board certified neuroradiologist (JBF).

### Neurological examination

The National Institute of Health Stroke Scale (NIHSS) is a validated instrument used in many clinical trials to assess neurological deficits of stroke patients [20]. The NIHSS displays the subjects' consciousness, the ability to move limbs, the sensorium, speech, vision and eye movements. The modified Rankin Scale (mRS) is used to quantify the persisting handicap after stroke [21].

### Neuropsychological examination

The neuropsychological assessment was designed to assess important cognitive domains and includes the following tests: Trail Making Test A and B (information processing and divided attention), the Stroop Test, the Category and Letter Fluency Test (all three assessing different aspects of executive functions) [22], the subtest 3 from the German Leistungsprüfsystem (reasoning) [23], a German version of the Rey Auditory Verbal Learning Test [24], the digit-span forward and backward test from the Wechsler Memory Scale - Revised [22] (verbal short-term and working memory), the German Mehrfachwahl-Wortschatz-Intelligenztest (MWT-A Test; educational level) [25] as well as the Rey-Osterrieth Complex Figure Test (visuo-spatial skills and visual memory) [22].

## Discussion

The pulmonary vein (PV) ablation in patients with symptomatic paroxysmal atrial fibrillation (AF) has received increasing attention over the last years and is now a well-established therapeutic approach to limit symptomatic paroxysmal AF [6,7]. The use of novel catheter techniques gives reasons to expect better safety and efficacy, but there is little clinical evidence so far [16,17]. Performing MACPAF, we hypothesize that the cryoenergy device (Arctic Front^®^) is superior to the balloon based device using radiofrequency (HD Mesh ablator^®^) with regard to efficacy and safety. Efficacy of LACA will be assessed by achieving a complete PV isolation as an acute effect, and by a lack of AF recurrence after a blanking period of three months (long-term efficacy), evaluated by an implantable device able to detect AF.

Focusing on the safety of LACA, the use of diffusion-weighted magnetic resonance imaging will precisely detect (silent) cerebral infarction, as recently demonstrated using a 1.5T MRI [15]. The use of a 3T MRI enables a higher resolution, improving the significance of stroke detection, while the detection of cerebral hemorrhage, chronic lesions and brain vessel occlusion is at least equal to 1.5T MRI [26]. Therefore, we expect a higher rate of silent strokes than the reported 10-11.3% [14,15]. Furthermore, the MACPAF study will demonstrate if the incidence of clinically silent or clinically evident strokes differs within the tested ablation systems. Moreover, a retrospective analysis of MACPAF might identify LACA procedure-independent risk factors, hopefully reducing LACA-associated stroke rate according to optimized technical conditions and patient selection. Using an implantable device for AF detection and a follow-up MRI after 6 months, we will be able to correlate the rate of (silent) strokes with the AF burden in patients on oral anticoagulation.

According to a http://www.clinicaltrial.org search, the MACPAF study meets highest technical standards. To eliminate potential conflicts of interest, this is an investigator-initiated clinical trial.

Nevertheless, the MACPAF study has its strengths and limitations. Firstly, using two balloon based ablation systems, our results are not transferable to other LACA procedures. Secondly, focusing on paroxysmal AF, we are unable to address questions regarding persistent AF. Thirdly, the assessment of the predefined endpoints is blinded but the LACA procedure itself can not be done in a blinded fashion. Fourth, the limited number of patients might not allow correction for multiple testing. Fifth, according to the study design, a placebo-arm is not intended. The lack of a placebo arm is justified as ablation is a clinically indicated procedure and thus withholding any ablation would be unethical. Prospective multicenter studies will be needed to clarify these important issues.

Taken together, MACPAF is a prospective randomized single-center trial to compare the efficacy and safety of LACA for paroxysmal AF using two balloon based catheter techniques. Results from MACPAF will provide important information helping cardiologists and neurologists to better counsel AF-patients concerning risks and benefits of LACA. Doing MACPAF, we hope to make a significant contribution to the evaluation of the LACA-associated (silent) stroke risk, setting the stage for stroke-risk reduction by improved technical conditions and appropriate patient selection.

## List of abbreviations

AF: atrial fibrillation; LACA: left atrial catheter ablation; MRI: magnetic resonance imaging; MACPAF: Mesh Ablator versus Cryoballoon Pulmonary Vein Ablation of Symptomatic Paroxysmal Atrial Fibrillation Study; PV: pulmonary veins.

## Competing interests

KGH reports lecture fees by Sanofi-Aventis and LK reports lecture fees by Medtronic. ME reports receiving consulting, lecture, grant and/or advisory board fees by BMS, Sanofi-Aventis, Boehringer-Ingelheim, Novartis, Pfizer and AstraZeneca. AS reports lecture fees and prior study grants by Medtronic and C.R. Bard. JBF reports receiving consulting, lecture and advisory board fees by BMS, Siemens, Philips, Perceptive, Bio-Imaging Technologies, Boehringer Ingelheim, Lundbeck and Sygnis.

## Authors' contributions

All authors have read and approved the final manuscript. LK, AS, KGH, ME, HPS and JBF initiated the study. LK and KGH wrote the study-protocol and the manuscript. LK will do the patient inclusion, JU the neuropsychological examination and KGH the neurological and the MRI examinations under supervision of JBF. Blinded MRI reading will be done by JBF. PUH did and will do the statistical calculations.

## Pre-publication history

The pre-publication history for this paper can be accessed here:

http://www.biomedcentral.com/1471-2377/10/63/prepub
